# Tat-induced histopathological alterations mediate hippocampus-associated behavioural impairments in rats

**DOI:** 10.1186/s12993-014-0047-3

**Published:** 2015-02-07

**Authors:** Rivona Harricharan, Veneesha Thaver, Vivienne A Russell, William M U Daniels

**Affiliations:** School of Laboratory Medicine and Medical Sciences, Discipline of Human Physiology, University of KwaZulu-Natal, Westville Campus, Private Bag X 54001, Durban, 4000 South Africa

**Keywords:** Tat protein Clade B, Morris water maze, Novel object recognition test, Histopathology, Hippocampus

## Abstract

**Background:**

HIV-1 is a global catastrophe, and is exceedingly prevalent in Sub-Saharan Africa. HIV-associated neurocognitive disorder is characterized by symptoms such as motor impairments, a decline in cognition, and behavioural irregularities. The aim of this study was to provide insight into the fundamental behavioural and histopathological mechanisms underlying the development and progression of HIV-1 neuropathology.

**Methods:**

Using stereotaxic techniques, Tat protein Clade B (1 μg/μl, 10 μl) was injected bilaterally into the dorsal hippocampus of male Sprague–Dawley rats. The Morris water maze (MWM) and novel object recognition test (NORT) were used to assess spatial learning and recognition memory, respectively. Haematoxylin and eosin staining was used to identify the histopathological changes.

**Results:**

A highly significant increase in latency to reach the hidden platform in the MWM implied that noteworthy hippocampal damage had occurred. Severe behavioural deficits were also observed in the NORT where the Tat-injected group showed a greater preference for a familiar object over a novel one. This damage was confirmed by the histopathological changes (increased astrogliosis, cells becoming eosinophilic and a significant reduction in the pyramidal cell layer) observed in the hippocampus. Additionally, increases in the hippocampal mass and protein were observed, consistent with the structural alterations.

**Conclusion:**

This study highlights the relationship between hippocampal-associated behavioural changes and histologic alterations following stereotaxic intra-hippocampal administration of Tat protein in rats. The implications of this study may positively impact the fields of immunology and neuroscience by encouraging future researchers to consider novel strategies to understand the complexities of the pathogenesis of HIV-associated neurocognitive disorder.

## Introduction

The prevalence of human immunodeficiency virus (HIV) infection remains a global calamity, particularly in South Africa where a prevalence rate of 4.6% has been reported (World Health Organization Statistics, 2013). Parallel to this concern is the escalating incidence of neurocognitive impairments observed in patients living with HIV. In a recent study Joska *et al*. (2010) reported that 23.5% of HIV-positive individuals screened at HIV clinics displayed some form of cognitive deficiency [1]. Comparable prevalence rates have been documented for HIV-positive populations elsewhere [2]. Despite this alarming statistic only a few studies have focused on investigating the effects of HIV on brain function. Nevertheless important data have been forthcoming the past two decades that have improved our understanding of the disease. For instance HIV-associated neurocognitive disorders may present with varying degrees of motor impairment (such as tremor, impaired movement and problems associated with speech) and a progressive decline in cognition (diminished memory function and difficulty associated with concentration) [3].

HIV-mediated dementia (HAD) has declined since the employment of highly-active antiretroviral therapy (HAART), however sparking a surge in the prevalence of HIV-associated neurocognitive disorders (HAND) [4]. Prior to 1991, HAD was the only neurocognitive disorder that had been defined. A refined and more accurate classification system for HAND was introduced in 2007 which categorized Mild Neurocognitive Disorder (MND) and Asymptomatic Neurocognitive Impairment (ANI) as two neurocognitive disorders in addition to HAD. Both MND and ANI involve loss within at least two cognitive areas however a patient with ANI does not display functional impairment which is observable. With the rise in the occurrence of MNI and ANI, the incidence of all cases of HAND is estimated to be 40-50% [4]. Previous studies have presented evidence that Tat protein is sufficient to mediate cognitive and behavioural abnormalities as seen in HIV-infected individuals and in animal models of the disease [5,6]. Bruce-Keller *et al.* has shown that genetically infusing C6 glioma cells into rat hippocampus elicited Tat production subsequently resulting in damage to neurons, impaired rotorod performance and increased gliosis [7]. Additionally a study involving human brains has shown that the hippocampus is one area particularly vulnerable to damage during HIV-infection [8]. Despite the high prevalence, the aetiology of the direct behavioural effects caused by Tat on learning and memory has not been fully elucidated.

The central nervous system acts as a reservoir for HIV [9]. Interestingly, viral load has been shown to be a poor indicator of the neuropathogenesis of HAND. Instead the condition appears to correlate better with the activation of glia following the transendothelial migration of infected macrophages into the brain [10]. Subsequent neuronal injury may therefore result either directly or indirectly from HIV-infected astrocytes and microglia [3,9,11]. Astrocytes have the ability to release harmful compounds (viral or cellular) or may influence neighbouring cells (like microglia) to produce harmful compounds which may damage neurons [4,12,13]. Whether these responses of astrocytes and/or microglia cause the deterioration in cognitive function associated with HIV infection, remains uncertain.

The HIV genome includes three structural genes (gag, pol, env), four accessory genes (vif, vpr, vpu, nef) and two regulatory genes (tat and rev) [14]. HIV-1 Tat (Transactivator of transcription) is a multifunctional protein in that it suppresses DNA polymerization, promotes tRNA placement onto HIV RNA, alters chromatin structure, phosphorylates RNA polymerase II and is responsible for viral gene transactivation [14]. Tat has also been shown to be toxic to neurons [15-17]. Consequently this viral protein has been implicated in instigating the behavioural changes that are observed with HIV infection. However evidence to support this notion has been limited [5].

Humans usually access memory through the medium of language (written, spoken, conceptual). However in order to understand the effect of Tat protein on the central nervous system (CNS) of animals, cognition must be assessed through the variety of behaviours they exhibit in response to different experimental paradigms, testing their learning performance and memory capacity [18]. Several brain regions like the hippocampus are stimulated in tests of memory and learning. The hippocampus was discovered to play an indispensable role in spatial memory in rats [19]. The Morris water maze (MWM) and the novel object recognition test (NORT) are widely accepted tests of spatial learning and recognition memory, respectively, in rats [18,20]. Since the NORT does not employ positive (food) or negative reinforcement (shock), it is considered to produce results that are comparable to memory tests in humans [20]. Elimination of positive and especially negative reinforcement minimizes stress of the animal and also removes confounding factors, thereby improving accuracy and reliability of the data [20].

The aim of the present study was therefore to provide further insight into the fundamental effects of Tat on the central nervous system and to explore it as a possible aetiological factor in the development of HIV-associated neurocognitive disorder. We specifically focused on the histopathological consequences of Tat injected directly into the dorsal hippocampus of rats. The effects of the Tat injection on the cognitive function of the animals were also investigated.

### Experimental procedures

#### Animals

A total of 25 adult male Sprague–Dawley rats (250-300 g, approximately 2–3 months old) were used in this study. To limit the confounding factors affecting our study, male Sprague–Dawley rats were used to avoid the effect of female hormones on the cells in the brain due to the fact that during pro-oestrus, there is an increase in cell proliferation which results in a greater number of immature neurons in the hippocampus [21] which would have affected the cellular morphology in our micrographs. Furthermore, during pro-oestrous, dendritic spine density is at its highest levels while the lowest levels are observed in the oestrous phase [22]. Since these changes were observed in the CA1 region of the hippocampus, one of the areas of interest in the present study, they could potentially have affected our results. In a study by Korol *et al.* (2004), the authors claimed that natural fluctuations of ovarian hormones can introduce bias in the neural system causing it to favour certain cognitive strategies [23]. During the pro-oestrous phase, rats were likely to implement place strategies as opposed to the estrous phase, where the rats favoured response strategies.

The animals were obtained from the Biomedical Research Unit at the University of KwaZulu-Natal and housed in this facility under standard laboratory conditions of 23 ± 1°C, 70% humidity and a 12 h light–dark cycle with lights on at 06 h00. The rats received food (rat chow) and water *ad libitum*. Ethical approval for the study was obtained from the Biomedical Research Ethics Committee (Ethics approval number 067/11). Rats were randomly divided into 2 groups namely a control group and an experimental group. The cognitive abilities of all the animals were assessed by subjecting them to behavioural tests (MWM and NORT) post injection of either Tat protein or saline into their hippocampi.

#### Study design

The first 3 consecutive days (Day 1–3) were allocated to MWM pre-injection training trials, followed by a test on Day 4. On Day 5, the control animals received bilateral 0.9% Saline injections (10 μl) into their dorsal hippocampi, while the experimental animals were treated with Tat (1 μg/μl, 10 μl). The animals were allowed to recover over the next 3 days (Days 6–8). To determine the effect of the intra-hippocampal administration of Tat on behaviour, post-injection trials were conducted for both groups of animals. The post-injection MWM test 1 and test 2 being performed on Day 9 and 12 respectively. Object familiarization for the NORT occurred from Day 13–15 with the NORT conducted on Day 16. The animals were sacrificed on Day 17.

#### Stereotaxic surgery

Recombinant Tat Clade B was obtained from *Diatheva (Milan, Italy).* Using stereotaxic techniques, Tat protein Clade B (1 μg/μl, 10 μl) was injected bilaterally into the dorsal hippocampus of the animals at the following coordinates: −3.7 mm posterior to bregma, ± 2.6 mm from the midline and 3.2 mm ventral to the surface of the skull [24]. This concentration of Tat protein was chosen since Gavriil *et al*. (2000) showed that injecting 20 μg of Tat protein into the striatum was sufficient to induce apoptosis [25]. Control animals were subjected to the same procedure where they received an equal volume of 10 μl physiological saline (which served as the vehicle) instead of Tat protein.

The rats were anaesthetized by injecting a standard combination of pentobarbital and atropine at a dose of 60 mg/kg and 0.2 mg/kg respectively. After the surgery, all animals were allowed to recover for 3 days before the commencement of post-injection behavioural tests. Two animals died during/after surgery (1 from the Saline-injected group and 1 from the Tat-injected group).

Twelve days after the intrahippocampal injections, six animals (three from the Saline group and three from the Tat group) were anaesthetized and subjected to transcardial perfusion with 4% paraformaldehyde in phosphate buffered saline. Following perfusion, the brains were harvested and stored in formalin for a minimum of 48 hours, to ensure adequate fixation.

### Behavioural tests

#### Morris Water Maze (MWM)

**Figure 1:**
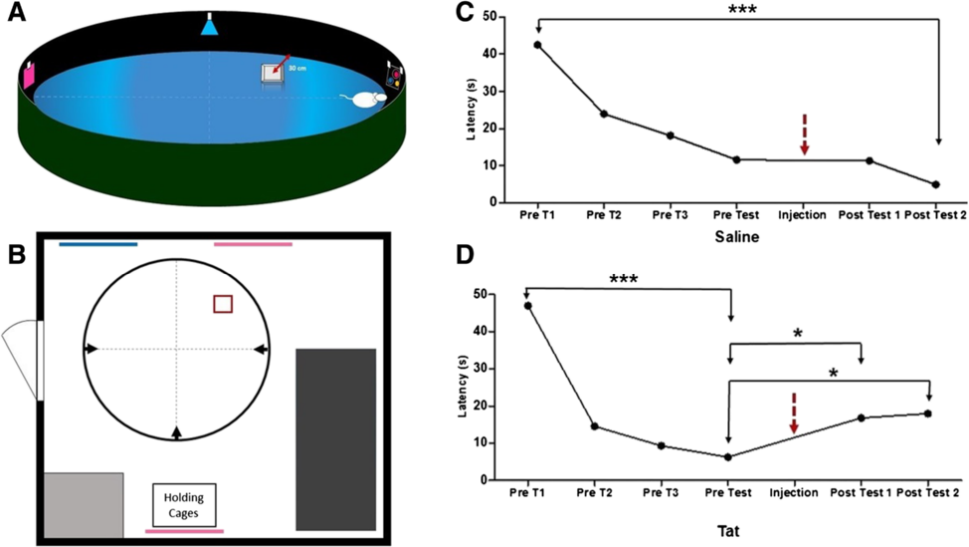
**The MWM room design and the performance of the Saline-injected rats and Tat-injected rats in the MWM. (A)** A 3-dimensional representation indicating the position of the platform in the MWM. **(B)** A simplified schematic diagram indicating the layout and the position of the visual cues present in the room where the pool was located (Pink and Blue lines represent proximal and distal visual cues, the light grey and dark rectangles represent a sink and table respectively, and the small square represents the position of the platform). Arrowheads represent the different release points; **(C)** The decreasing latency in the Saline-injected rats persisted from pre-injection to post-injection; **(D)** Latency to find the platform in the MWM was impaired in Tat-injected rats (Results are mean ± SEM). * Post-injection Tat significantly different from pre-injection Tat, p < 0.05. *** Pre-injection trial 1 significantly different from Pre-injection test for the Tat group, p < 0.0001. (Saline: n = 9; Tat: n = 14).

The MWM assesses the learning and recollection ability of rats involving the use of exploratory, navigational, spatial and contextual memory [26]. The MWM consists of a hidden platform located in one of the quadrants of a 1 m diameter circular pool with a height of 85 cm (Figure 1A). The platform was constructed of transparent plastic (11 × 11 cm and a height of 18 cm). The pool was filled with tepid water (27 ± 1°C). There were fixed visual cues attached to the walls of the pool as shown in Figure 1A. In addition, distal cues were represented by coloured sheets of paper attached to the walls of the room as indicated by the pink and blue lines in Figure 1B. The method entails placing the rat in a quadrant other than where a hidden platform is located and then the time taken (latency) for the rat to find the hidden platform is recorded. Each rat was released from three different quadrants and was allocated 120 seconds to find the platform. If they were unable to do so, the rat was then guided to the platform and allowed to remain on the platform for 15 seconds. The rats participated in three consecutive days of training pre-injection and the actual pre-injection test was conducted on the fourth day with the platform in the same place as during the trial days. Post-injection test one and two was conducted four and seven days after the injection respectively. For the post-injection tests the platform was still located in the same position as it was prior to the injection. In theory, the control animals would learn the position of the hidden platform quickly and hence they would need a short time period to locate the hidden platform. In contrast, cognitively impaired rats would be expected to take longer to learn how to navigate to the hidden platform and therefore they would require a longer time to locate it.

#### Novel Object Recognition Test (NORT)

**Figure 2:**
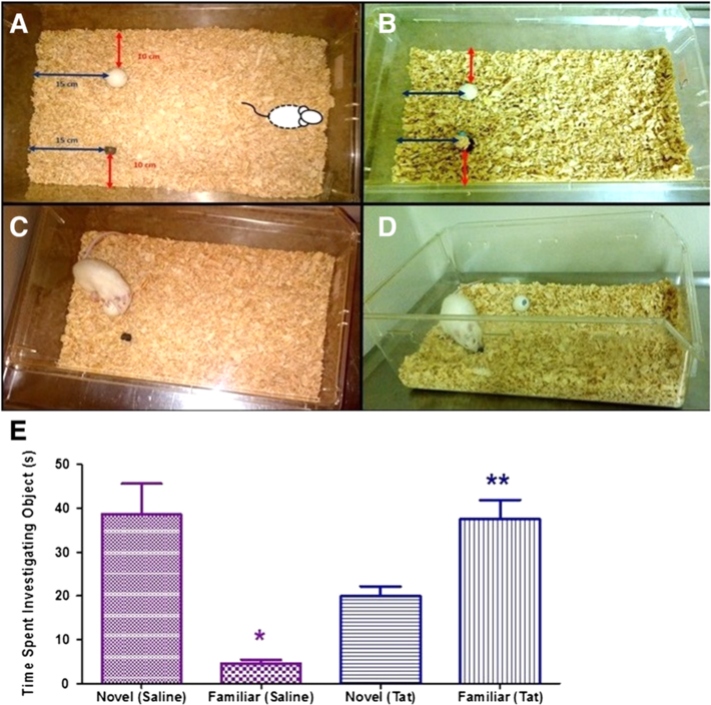
**The arena and performance in the NORT of the Saline-injected rats and Tat-injected rats. (A)** The novel object test arena displaying the familiar objects. **(B)** the novel object test arena displaying the novel object and one familiar object; **(C)** subject showing interest in the familiar object; **(D)** subject showing interest in the novel object; **(E)** graphical representation of the time spent with the novel and familiar objects by the saline- vs. Tat-injected groups. (Results are mean ± SEM). * Novel (Saline) significantly different from Familiar (Saline), p < 0.001. ** Novel (Tat) significantly different from Familiar (Tat), p < 0.01. (Saline: n = 9; Tat: n = 14).

The NORT was conducted to assess the rat’s ability to recognize a novel object as determined by their exploratory behaviour. A normal rat’s curious nature should cause it to display preference for exploring a novel object over a familiar object. A large cage was used as the arena for the test (52.5 cm × 35 cm). The familiarization of animals to the experimental procedures occurred on the first 3 days. On day 4 the test was done. On each familiarization day the animals were first exposed to the empty cage for 5 minutes, i.e. no objects present. The animals were thus allowed to become familiar with the cage environment. The animals were then removed for a brief period, during which the two similar objects were strategically placed according to Figure 2A. The removal of animals from the cage was deemed important so as not to attract their attention to the placement of the objects. The animals were given 10 minutes to explore these objects. During this period we observed that the animals were only “interested” in the objects in the first 2–3 minutes of exposure. On the following day, the recognition test was done, involving one of the familiar objects being exchanged for a novel object. This was placed in the exact location as its predecessor (Figure 2B). The time that each rat showed interest in the objects (approaches and physical exploration), was only recorded for the first 150 seconds (2.5 minutes). We defined ‘investigating an object’ as referring to the animal in close proximity to the object (<2 cm) and showing a direct interest in the object (meaning the animal interacting directly with the object such as sniffing, pawing, licking, moving or holding the object). We did not regard instances where the animal walked past the object or was in the vicinity of the object but not engaged in one of the previously mentioned behaviours.

### Histology

#### Haematoxylin & eosin

Three brains from the Saline group and three brains from the Tat-injected group were collected for histological analysis. The fixed brain was dehydrated, cleared with xylene and infiltrated with paraffin. The tissue was sectioned from a block approximately 6 mm thick with the site of needle entry in the middle. The block was trimmed and collection of sections occurred approximately 2 mm on both sides of the site of the injection. The wax-embedded brain tissue was then sectioned coronally into 3 μm sections using the rotary microtome, Microm HM315. The slices were collected on glass slides and immersed in increasing concentrations of alcohol solutions (80%, 90% and absolute alcohol twice) prior to being exposed to standard haematoxylin-eosin staining techniques for 10 minutes. The slides were subsequently placed in decreasing concentration of alcohol and finally mounted in DPX permanent mount. Photomicrographs of the slides and measurements of the pyramidal cell layers were obtained using the Leica SCN400 slide scanner (SMM Instruments, South Africa).

### Protein determination

The animals were decapitated, the dorsal hippocampus harvested and their wet weight recorded. The tissue was stored temporarily in liquid nitrogen for subsequent neurochemical analysis. Protein determination was done according to the Bicinchoninic acid Assay (BCA) a modification of the Lowry procedure. Briefly the hippocampal tissue samples were thawed and homogenized in 600 μl RIPA buffer using a sonicator. Following this procedure, the samples were centrifuged at 8000 g for 10 minutes at 4°C. The supernatant was extracted and then further centrifuged again to ensure the samples were clear. 50 ml of bicinchoninic acid solution (reagent A) was mixed with 1 ml of Copper (II) sulfate pentahydrate 4% solution (reagent B) to make up the BCA working reagent. Bovine serum albumin was used as the protein standard. The actual procedure entailed the addition of 2 ml of the BCA Working Reagent to 0.1 ml of each BSA protein standard and unknown sample. The standards and samples were gently vortexed and allowed to incubate at 37°C for 30 minutes. 100 μl of each standard and unknown sample was immediately transferred to a 96-well plate and the absorbance was measured at a wavelength of 562 nm.

### Statistical analysis

GraphPad Prism 5 (*Graph Pad Software Inc., USA*) was used to perform statistical analysis of the data where a p value < 0.05 was considered statistically significant. Tests for normality were conducted using the D’Agostino & Pearson omnibus normality test. The MWM data were found to have a non-Gaussian distribution. Subsequently non-parametric methods were used to analyse this data. Differences within the same group were assessed with the Kruskal-Wallis test followed by Dunn’s Multiple Comparison post-hoc test. Differences between the Saline-injected and Tat-injected groups was analysed using independent t-tests. The NORT data were analysed using one-way ANOVA with Bonferroni post-hoc test. Independent t-tests were used to analyse changes in the thickness of the pyramidal cell layer for the Saline-injected (n = 3) and Tat-injected (n = 3) groups. The hippocampal mass and protein levels were also analysed using independent t-tests (Saline: n = 6, Tat: n = 9). Data are presented as the mean ± standard error of the mean (SEM).

## Results

Kruskal-Wallis test and Dunn’s Multiple Comparison post-hoc test revealed significant differences in the latency to find the platform between pre-injection and post-injection tests in the Tat-injected group. Latency to find the platform decreased post-injection in the Saline-injected rats (Figure 1C). Latency to find the platform increased post-injection in the Tat-injected group (Figure 1D). Analysis of the data obtained in the MWM prior to the injection of saline or Tat revealed no statistically significant difference between the latencies of the Saline-injected and Tat-injected groups. However, analysis of the latencies to reach the hidden platform post injection showed that there was a statistically significant difference between the latencies of the Saline-injected and Tat-injected rats (p < 0.05).

One-way ANOVA revealed a significant difference in time spent exploring the novel object compared to the familiar object *(F*_*(3,42)*_*=14.07; p < 0.05)*. Saline-injected rats spent more time with the novel object than with the familiar object (p < 0.001) while Tat-injected rats spent less time with the novel object than with the familiar object (p < 0.01, Figure 2E). Based on the results depicted in Figure 2E, animals within the Saline group demonstrated a preference for the novel object over a familiar one. In contrast, the Tat-injected group showed greater preference for the familiar object by spending more time investigating the familiar object than the novel object as shown in Figure 2E. Saline-injected rats spent a greater amount of time investigating a novel object than the Tat-injected group (p < 0.05). The Tat-injected rats spent more time with a familiar object than the Saline-injected group (p < 0.001).

**Figure 3:**
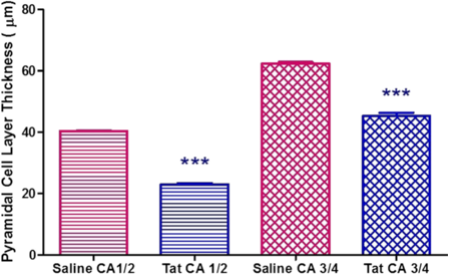
**Graphical representation showing the changes in the pyramidal cell layer in the CA 1/2 and CA 3/4 regions in the Saline-injected and Tat-injected groups (Results are mean ± SEM).** *** Tat is significantly lower than Saline, p < 0.001. (Saline: n = 3, Tat: n = 3).

**Figure 4:**
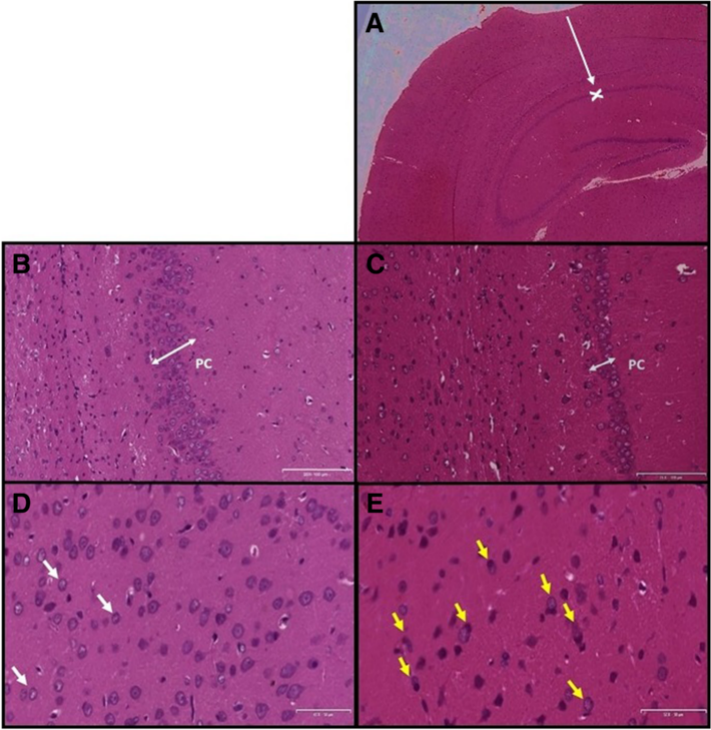
**Photomicrograph of haematoxylin and eosin stained Saline and Tat sections.** Photomicrograph of haematoxylin and eosin stained Saline and Tat sections **(A)** hippocampal formation and dentate gyrus of a Tat-injected brain (200×). White arrow represents the path of the tract created by the needle and the ‘x’ indicates the site of injection, Scale bar = 200 μm; **(B)** the pyramidal cell layer (PC) in a Saline-injected rat brain Post-injection (100×), Scale bar = 100 μm; **(C)** the pyramidal cell layer (PC) in a Tat-injected brain Post-injection (100×), Scale bar = 100 μm; **(D)** Neurons and glial cells in the Saline-injected brain (50×); White arrows represent normal astrocytes, Scale bar = 50 μm; **(E)** Neurons and glial cells in the Tat-injected brain (50×). Yellow arrows show astrocytes undergoing gliosis, Scale bar = 50 μm.

We observed a statistically significant reduction in the pyramidal cell layer thickness (particularly in CA 1/2 and CA 3/4 regions) of the hippocampus of the Tat-injected rats in comparison to the Saline-injected group. This analysis was conducted using independent t-tests to analyse the data (p < 0.001, Figure 3). Figure 4A-E shows the histological differences between the Saline-injected and Tat-injected rat hippocampus. The Tat-injected hippocampi exhibited darker staining in comparison to the Saline-injected group. Gliosis was also observed in the Tat-injected hippocampal tissue. The astrocytes in the Saline-injected group had discernible “naked” nuclei whereas the Tat-injected group showed reactive astrocytes (which were considerably larger in size) with more prominent hyperchromatic nuclei that had been displaced to the periphery.

**Figure 5:**
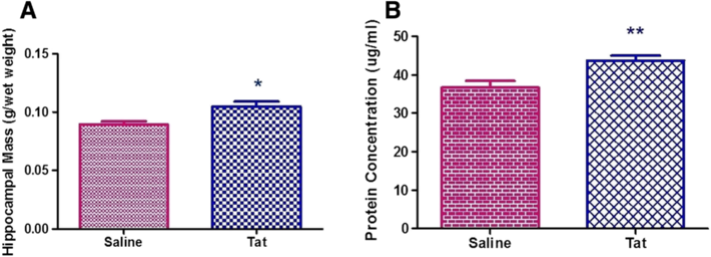
**The hippocampal mass and protein concentration of the Saline and Tat groups.** Graphical representation of **(A)** hippocampal mass of the Saline-injected and Tat-injected groups. **(B)** hippocampal protein of the Saline-injected and Tat-injected groups (Results are mean ± SEM). * Saline is significantly lower than Tat, p < 0.05, ** p < 0.01. (Saline: n = 6, Tat: n = 9).

Independent t-tests showed that the hippocampal mass of the Tat-injected rats was significantly greater than that of the Saline-injected rats (p < 0.05, Figure 5A). This was corroborated by the increase in hippocampal protein concentration of the Tat-injected rats compared to the Saline-injected rats (p < 0.01, Figure 5B).

## Discussion

Despite the administration of antiretroviral therapy, many HIV-infected individuals go on to develop cognitive impairments [27]. The likelihood of these alterations in brain function stemming from HIV is supported by the fact that the brain serves as a reservoir for the virus during latent periods [9]. We hypothesized that the viral protein Tat and not the intact virus may be adequate to induce cognitive abnormalities in mammals. To test this hypothesis we injected Tat directly into the hippocampi of rats and assessed the impact thereof on the cognitive behaviour of the animal. The brain tissue in the area of the injection sites was also histologically analysed to determine the effect of Tat on cellular morphology.

The Saline-injected group displayed normal learning patterns both pre- and post-hippocampal injections. This is consistent with results obtained for a Saline-treated group in a study by Meilandt *et al*. (2004) where their Saline-treated group displayed learning curves similar to Saline-injected rats in the present study [28]. The decrease in latency to find the platform in the second post-injection test of the Saline-injected group demonstrated their ability to learn and re-call the task more quickly than in the pre-injection test, accomplishing the task in their fastest times post-injection. This confirmed that the mechanical damage caused by administration of saline did not impair the learning-associated function of the hippocampus. The Tat-injected group showed normal learning curves prior to injection depicting the animal’s ability and mental capacity to learn the task and effectively exploit spatial and navigational cues allowing them to complete the task. Assessment of post-injection learning and recollection of the task showed that Tat significantly impaired the functioning of the hippocampus. The latency of the Tat-injected group to reach the platform markedly increased after the injection, demonstrating that intra-hippocampal administration of Tat directly affected the rat’s learning ability and performance of a spatial learning acquisition task. These results are in agreement with those reported by Fitting *et al.* (2008a, b) who showed that intrahippocampal injections of Tat on postnatal day 1 impaired spatial memory and decreased the number of neurons in the dentate gyrus at 7 months of age, while increasing the number of astrocytes and oligodendrocytes [29,30]. More recently, Fitting *et al.* (2013) also showed that Tat transgenic mice demonstrated an increased latency to find the hidden platform in the MWM [31]. Learning and memory are two functions that are innately associated with the dorsal hippocampus of the rat. Many studies have shown that damage to the hippocampus leads to deficits in learning and memory [32-35]. Our results therefore confirm these learning impairments. While several studies [36-38] including our study have placed emphasis on decreasing escape latencies as a measure of learning, it should also be noted that even blind animals may display decreased latencies [39]. Path length (which highlights searching behaviour) is also an important factor to consider.

Animals within the Saline-injected group displayed a preference for the novel object after three days of exposure to a familiar object. This is in agreement with published results involving mice [5]. In contrast, the Tat-injected group spent greater exploratory time investigating the familiar object in comparison to the novel object. Our findings are in agreement with a study by Torres & Noel (2014) who demonstrated that rats infected with HIV-1 viral protein R (Vpr) spent greater time exploring a familiar object over a novel one [40]. According to Ennaceur (2010), experimental rodents may experience difficulty in remembering the familiar object and perceive the familiar object as “novel” or “vaguely familiar”. This results in the experimental animals spending more time exploring the familiar object as opposed to the control animals who recognize the familiar object as being recently encountered [41]. Our results further suggest that the hippocampal impairment was due to the effect of the Tat and not as a result of mechanical damage caused by the needle tract or the procedure of the actual intrahippocampal injection itself. Similar to the present findings, Antunes & Biala, (2012) found that animals with hippocampal damage unintentionally express their preference for a familiar object rather than a novel object due to their reduced cognitive skills to recognize the novelty of the new object introduced [18]. Due to the great importance for rats to exhibit exploratory behaviour and their curious nature, normal and healthy rats would display a greater preference for a novel object.

In a recent clinical study Ortega *et al*. (2013) used neuropsychological tests and neuroimaging techniques to demonstrate a significant reduction in the volume of brain areas of HIV-positive patients that included the hippocampus, when compared to age-matched controls [42]. The decreases in brain volumes correlated well with the poor performances of the infected patients in the neuropsychological tests [42]. Comparably, we measured the weights and protein content of the hippocampi of the two groups of animals. We found that in contrast to expectation the Tat-injected hippocampi had a greater mass and total amount of protein in comparison to the Saline-injected group. A number of studies have shown that tat injected directly into the brain leads to various neurochemical abnormalities [15-17]. These included Tat-mediated NMDA receptor activation, dysregulation of calcium homeostasis, generation of reactive oxygen species, and activation of death-inducing signalling pathways with the initiation and execution of apoptotic cell death. With effects such as these one would expect Tat toxicity to lead to a reduction in protein concentration and tissue mass. However this was not the case in our study. A probable mechanism for the increase in protein content may involve the ubiquitin-proteosome system. Through protein ubiquitination damaged intracellular proteins are recognized and degraded to remove any potential danger for the cell. However the ubiquitin-proteosome system may also serve as a regulator of gene expression through the controlled destruction of cyclins, protein kinases and transcription factors. Production or accumulation of protein aggregates leads to disruption of the ubiquitin-proteosome system when these aggregates are deposited into cytoplasmic inclusion bodies [43]. Protein aggregate accumulation is a globally-recognized feature of several hereditary and sporadic neurodegenerative diseases [44], substantiating the hypothesis that the aggregation is an underlying molecular mechanism in neurodegenerative disease pathogenesis [45]. Ubiquitination has been shown to stimulate the transcriptional properties of Tat protein and ubiquitin plays a non-proteolytic function following its fusion to Tat protein [46]. This process may have accounted for the increase in protein content.

The increase in protein content may have disturbed proteostasis (protein homeostasis) within the endoplasmic reticulum (ER) leading to impaired production, folding or degradation of proteins. This occurs via the unfolded protein response (UPR) pathway. The role of the adaptive UPR pathway is to ensure that the ER is functioning adequately, while the apoptotic UPR pathway functions to exterminate cells subjected to chronic or rigorous ER stress. Dysregulation of the UPR system instigates the occurrence of oxidative stress consequently resulting in the induction of apoptosis. Based on the histopathology, we observed pyknotic nuclei, a common characteristic of apoptosis. It is therefore not surprising that dysfunction of the UPR system leading to the loss of pyramidal cells via apoptosis may have occurred in our Tat-induced rat model of HAND because of the emerging importance of improper functioning of the UPR system in the development of neurodegenerative diseases [47].

Histological analysis of the hippocampi of the Saline-injected group displayed features of a healthy, normal hippocampal formation whereas the Tat-injected group showed distinct differences. The pyramidal cells and areas of the hippocampus located near the dentate gyrus in the Tat-injected group appeared darker in the photomicrograph as compared to that of the saline-injected group. A possible explanation for this difference may stem from glial cells (like astrocytes) undergoing gliosis. During this process the pH of the cellular microenvironment decreases to become more eosinophilic. This lower pH increases the cell’s affinity for the haematoxylin and eosin stain and may lead to the darker colour intensity [48]. Normal astrocyte cytoplasm is usually discernible with haematoxylin and eosin staining. The Tat-infected brains displayed reactive astrocytes with more prominent hyperchromatic nuclei which had been displaced to the periphery. This finding was consistent with the observations of Aschner & Costa (2004) who described the cytoplasm of astrocytes to become more distinct by 7–10 days following an insult [49]. Interestingly these authors also reported astrocyte proliferation in addition to its hypertrophy. Productive infection or infection by the whole virus is not required to induce alteration in the BBB. Tat can effectively be translocated into the CNS by means of the BBB [14]. Cytokines like TNF-α have been implicated in regulation of synaptic plasticity in neuropathological states subsequently resulting in memory and learning deficits [50]. This is consistent with the neurological deficits associated with HIV-mediated neurocognitive disorders. Tat elicits its secondary effects, commencing with cytokine production within the CNS which consequently results in glial activation first and then attracts the infiltration of monocytes into the brain [51]. TNF-α along with other cytokines enhance astrocytosis/astrogliosis, a process that occurs frequently in HIV-mediated neurocognitive disorders where there is an increase in the size and quantity of astrocytes [52]. Activated macrophages are attracted by astrocytosis [3]. Increased cytokine production in the periphery only occurs subsequently after the activation of glia. Fiala *et al.*, (1997) found that early after the infection, the brain is protected from HIV-1 by the BBB [51]. However cytokines like TNF-α open a paracellular route into the brain. Since there have been several studies involving the immune response and cytokine production initiated by Tat protein, this study elaborates on the behavioural and histopathological aspects which have not been fully understood.

Another prominent histological difference observed between saline-injected and Tat-injected brains was the thickness of the pyramidal cell layer. In saline-treated controls the pyramidal cell layer consisted of 5–6 cells in thickness (approximately 40 μm in the CA 1/2 region and 63 μm in the CA 3/4 region) forming distinct layers as opposed to the significant perturbation of the pyramidal cell layer in the Tat-injected brain where it only consisted of 2–3 cells in thickness (approximately 23 μm in the CA 1/2 region and 45 μm in the CA 3/4 region). This suggested that Tat caused a loss of pyramidal neurons, an observation that was in agreement with data of Kaul and Lipton (2006) who found that exposure to HIV-1 proteins does indeed cause extensive loss of pyramidal neurons [53]. Our results are further supported by Bell *et al*. (2003) who showed that an increase in gliosis is often associated with microtubule loss and subsequent neuronal loss in the CA3 region of the hippocampus and the dentate gyrus in animals following exposure to viral proteins of HIV [54]. Since Tat is the only viral protein known to be released by infected microglial cells [55] it may have accounted for the increased protein concentration as we have shown that active gliosis is occurring and this process occurs subsequently after activation of the macro- and microglia, however this is only our speculation. The fact that Tat-treated animals had greater hippocampal mass suggested that the increase in astrogliosis superceded the rate of neuronal loss at this stage of disease pathogenesis. We therefore speculate that while the degree of neuronal loss was insufficient to be reflected in atrophy of the hippocampus, it was adequate to induce impaired cognitive function.

## Conclusion

In this study, we have shown that direct injection of Tat protein into the hippocampus of rats may lead to learning and memory deficits in these animals. This impairment in cognitive behavior was associated with significant abnormalities in the microarchitecture of the hippocampus. Although several recent studies have shown Tat protein to be a promising candidate for a vaccine in HIV infection [56,57], we were interested in examining its adverse effects. In this particular study, we investigated the early stages of the HIV-1 infection but in future, we suggest that extending the time frame of the study could assist in better comprehension of the disease progression. The implications of this study may positively impact the fields of immunology and neuroscience. In the field of Neuroscience, the escalating volume of research continues to generate novel findings. These discoveries offer promising new targets for therapeutic interventions and possibly a way to reduce the damage caused by HAND. These novel findings only arise through comprehensive understanding of the neurological and immunological factors which influence the etiological mechanisms involved in HIV-related neurocognitive disorders.

## Abbreviations

ANI: Asymptomatic neurocognitive impairment
ANOVA: Analysis of variance
BBB: Blood brain barrier
BCA: Bicinchoninic acid assay
BSA: Bovine serum albumin
CA: Cornu ammonis
HAART: Highly-active antiretroviral therapy
HAD: HIV-associated dementia
HAND: HIV-associated neurocognitive disorder
HIV: Human immunodeficiency virus
MWM: Morris water maze
MND: Mild neurocognitive disorder
NMDA: N-Methyl-D-aspartate
NORT: Novel object recognition test
RNA: Ribonucleic acid
TNF-α: Tumor necrosis factor alpha
UPR: Unfolded protein response
Vpr: Viral protein R
